# Intrinsic Radiosensitivity and Cellular Characterization of 27 Canine Cancer Cell Lines

**DOI:** 10.1371/journal.pone.0156689

**Published:** 2016-06-03

**Authors:** Junko Maeda, Coral E. Froning, Colleen A. Brents, Barbara J. Rose, Douglas H. Thamm, Takamitsu A. Kato

**Affiliations:** 1 Department of Environmental & Radiological Health Sciences, Colorado State University, Fort Collins, CO, United States of America; 2 Department of Clinical Sciences, Colorado State University, Fort Collins, CO, United States of America; National Cheng Kung University, TAIWAN

## Abstract

Canine cancer cell lines have progressively been developed, but are still underused resources for radiation biology research. Measurement of the cellular intrinsic radiosensitivity is important because understanding the difference may provide a framework for further elucidating profiles for prediction of radiation therapy response. Our studies have focused on characterizing diverse canine cancer cell lines *in vitro* and understanding parameters that might contribute to intrinsic radiosensitivity. First, intrinsic radiosensitivity of 27 canine cancer cell lines derived from ten tumor types was determined using a clonogenic assay. The 27 cell lines had varying radiosensitivities regardless tumor type (survival fraction at 2 Gy, SF2 = 0.19–0.93). In order to understand parameters that might contribute to intrinsic radiosensitivity, we evaluated the relationships of cellular radiosensitivity with basic cellular characteristics of the cell lines. There was no significant correlation of SF2 with S-phase fraction, doubling time, chromosome number, ploidy, or number of metacentric chromosomes, while there was a statistically significant correlation between SF2 and plating efficiency. Next, we selected the five most radiosensitive cell lines as the radiosensitive group and the five most radioresistant cell lines as the radioresistant group. Then, we evaluated known parameters for cell killing by ionizing radiation, including radiation-induced DNA double strand break (DSB) repair and apoptosis, in the radiosensitive group as compared to the radioresistant group. High levels of residual γ-H2AX foci at the sites of DSBs were present in the four out of the five radiosensitive canine cancer cell lines. Our studies suggested that substantial differences in intrinsic radiosensitivity exist in canine cancer cell lines, and radiation-induced DSB repair was related to radiosensitivity, which is consistent with previous human studies. These data may assist further investigations focusing on the detection of DSB for predicting individual response to radiation therapy for dogs, regardless of tumor type.

## Introduction

Cancer is a major cause of death in dogs as well as in humans. Human and canine cancers have similar characteristics, not only in anatomical and histopathological appearance but also biological behavior, tumor genetics and response to conventional therapies [1, 2]. Canine cancer models have emerged as valuable resources in the study of human cancer [2]. In human cancer research, numerous well characterized human cancer cell lines are available for cancer research. Cancer cell lines have been widely used as *in vitro* experimental model systems and have proved to be useful for exploring the underlying biology of cancer [3]. Canine cancer cell lines have progressively been developed and utilized, but are not as fully characterized as human cell lines. Investigation of the cellular biology through characterizations of canine cancer cell lines may provide additional information about cancer biology, some specific to dogs, and some potentially supplementing those reported for human cancer.

Tumors even with same histopathological origin may show a wide range of sensitivity to radiation therapy [4, 5]. Measurement of cellular intrinsic radiosensitivity is important because understanding the difference may provide a framework for further elucidating profiles for prediction of radiation therapy (RT) response. Intrinsic radiosensitivities measured by *in vitro* colony formation assays are expressed as SF2, the fraction of cells surviving a single 2 Gy dose of ionizing radiation (IR). The dose of 2 Gy is also a commonly used dose per fraction in clinical RT in humans. The SF2 in humans has been shown to predict tumor response *in vivo* in previous studies [6, 7]. Such studies have suggested that differences in intrinsic radiosensitivity exist and understanding the mechanisms could significantly impact practice for personalized RT [4, 5].

The mechanisms underlying the differences in intrinsic radiosensitivity of tumor cells is likely multifactorial [5]. Repair of DNA double strand breaks (DSBs) is known as one of the most important elements that determines intrinsic radiosensitivity because these lesions, if unrepaired, lead to cell death [8]. Previously, the distribution of the cells in the phases of the cell cycle and DNA/chromosome content have been suggested as factors which may affect intrinsic radiosensitivity of tumor cells [9, 10]. Furthermore, part of the differences might be attributable to the tendency to undergo apoptosis in response to radiation as seen in lymphoid tumors [11]. However, inconsistent correlations with radiosensitivity of human tumor cells have been reported in the measurement of these parameters, and establishment of a useful assay that predicts intrinsic radiosensitivity is still under investigation [4].

Our studies have focused on characterizing diverse canine cancer cell lines *in vitro* and understanding parameters that might contribute to intrinsic radiosensitivity. This basic characterization can provide information of these cell lines for further research in prediction of radiotherapy response. We examined the intrinsic radiosensitivity of 27 canine cancer cell lines derived from ten tumor types. Each cell line was characterized by a combination of data representing cell cycle distribution, cellular doubling time, chromosome number, DNA ploidy pattern and plating efficiency. The known parameters including DNA DSB repair efficiency and apoptosis following ionizing radiation exposure were evaluated between selected radiosensitive and radioresistant cell lines.

## Materials and Methods

### Cell Culture

The 27 canine tumor cell lines were kindly supplied by Flint Animal Cancer Center of Colorado State University (Fort Collins, CO, USA) (Table 1) [12]. Adhesive tumor cell lines were grown in Minimum Essential Medium (MEM/EBSS, Thermo Fisher Scientific, Waltham, MA) supplemented with 10% fetal bovine serum (FBS; Sigma-Aldrich, St Louis, MO), 1% MEM vitamins, non-essential amino acids, sodium pyruvate, penicillin, streptomycin and Fungizone. Suspension tumor cells were grown in RPMI 1640 medium (Gibco Life Technologies) supplemented with the same as the MEM. Cell lines were maintained at 37°C in a humidified incubator with 95% air and 5% CO_2_.

**Table 1:** Characteristics and radiation survival of 27 cell lines used in this study.

			Cell cycle distribution (%)								
Cell line	Type	DT (hr)	G1	S	G2/M	Relative DNA content 2n = 1^b^	No. of chromosomes/cellMean ± SD	X-shape^c^ (%)	PE	SF2	SF5
D17^**a**^	OSA	22	43.3	28.1	28.5	1.4	79.1±25.4	54	0.24	0.70	0.25
Abrams	OSA	19	49.0	36.8	14.4	1.6	108±21.0	15	0.43	0.85	0.50
Moresco^**a**^	OSA	22	67.5	28.3	4.20	1.6	82.4±15.9	38	0.29	0.81	0.38
Gracie*	OSA	19	44.4	38.6	17.0	1.1	74.3±4.8	5.8	0.23	0.77	0.33
MacKinley^**a**^	OSA	28	40.2	47.7	12.1	1.0	58.2±5.1	27	0.22	0.44	0.04
HMPOS	OSA	20	52.7	35.4	12.0	1.6	84.3±10.6	35	0.23	0.80	0.29
OSA8	OSA	21	67.5	22.8	9.83	1.5	94.5±6.70	15	0.34	0.84	0.33
17CM98	Melanoma	22	62.0	22.7	15.3	2.1	155±28.5	1.5	0.23	0.67	0.20
CML-6M	Melanoma	15	51.9	34.3	13.8	1.0	78.6±5.24	2.6	0.61	0.74	0.27
CML-10C2	Melanoma	21	50.7	32.2	17.1	1.4	105±16.6	6.3	0.19	0.38	0.02
Jones	Melanoma	14	55.0	33.5	11.5	1.2	81.0±3.61	2.5	0.62	0.66	0.15
Parks	Melanoma	30	42.3	17.3	29.9	1.2	73.7±11.5	3.8	0.15	0.72	0.17
CMT-12	Breast	19	37.7	39.4	22.9	2.0	121±20.3	13	0.43	0.93	0.69
CMT-27	Breast	18	42.1	35.2	22.8	1.1	76.1±20.5	13	0.50	0.86	0.39
DEN-HSA	HSA	16	47.7	36.5	15.7	1.2	76.9±6.67	6.9	0.44	0.86	0.40
K9TCC	Bladder	18	46.7	37.4	15.9	1.0	77.3±14.3	11	0.54	0.90	0.60
Bliley	Bladder	20	55.1	28.7	16.2	2.1	145±31.8	3.6	0.16	0.44	0.04
DH82	Histiocytic	26	45.8	31.7	22.6	1.6	72.2±13.4	39	0.35	0.69	0.10
Nike	Histiocytic	25	42.1	37.9	20.0	1.4	64.4±102	30	0.14	0.19	0.01
STSA-1	Soft-tissue	40	42.3	27.8	29.9	1.0	60.5±12.1	26	0.22	0.44	0.12
CTAC	Thyroid	15	53.2	36.7	10.2	1.2	81.1±13.4	9.6	0.54	0.80	0.26
1771	Lymphoid	24	66.2	28.5	5.38	1.1	56.6±3.02	34	0.65	0.75	0.27
OSW	Lymphoid	16	56.2	36.6	7.28	1.0	73.6±2.56	2.6	0.44	0.61	0.15
CLBL1	Lymphoid	19	39.3	50.1	10.5	1.0	73.1±15.2	6.5	0.37	0.53	0.06
CLL1390	Lymphoid	37	62.6	33.6	3.74	1.6	143±29.2	4.8	0.63	0.85	0.36
C2	Mast cell	21	64.7	27.1	8.18	1.5	94.0±9.39	17	0.45	0.64	0.25
BR	Mast cell	36	69.6	28.9	1.52	0.9	73.5±7.05	4.5	0.04	0.56	0.10

Abbreviations: OSA, osteosarcoma; HSA, hemangiosarcoma; DT, doubling time; PE, plating efficiency; SF2, survival fraction at 2 Gy; SF5, survival fraction at 5 Gy.

^a^Data from Maeda et al., 2012

^b^The diploid amount of DNA (2n) is 1.

^c^Percentage of metacentric chromosome.

### Cell Proliferation

In order to determine the doubling times of the cell lines, cells were plated at different concentrations in 35 mm culture dishes. Cells were incubated at 37°C. The number of cells was counted every 24 hours using a Coulter counter Z1 (Beckman Coulter, La Brea, CA). Cellular doubling times were calculated using Prism 5 software (Graph Pad Software, La Jolla, CA). At least three independent experiments were carried out.

### Chromosome Number

Cells were cultured with 0.1 μg/mL colcemid (Invitrogen, Carlsbad, CA) for 6 hours in order to harvest metaphase chromosomes. Samples were treated in hypotonic 75 mM KCl solution for 20 minutes at 37°C and fixed in 3:1 (methanol: acetic acid) fixation solution three times. Metaphase spreads were stained with Giemsa solution, and the chromosome number was observed under an Axioplan microscope (Carl Zeiss, Jena, Germany). A minimum of 100 metaphase cells were analyzed to count chromosomes per cell. At least 50 metaphase cells were analyzed to count metacentric chromosomes per cell.

### Cell Cycle Analysis

Cell cultures at 60% to 70% confluence were fixed in 70% ethanol at -20°C overnight or longer. Cells were then centrifuged at 1,500 rpm for 5 minutes and washed once with PBS. Cells were then resuspended in 1 mL of staining solution (20 μg/mL propidium iodide, 0.1% TritonX-100, 500 μg/mL RNase A) and incubated for 30 minutes at room temperature. Analysis was done using a FACSCalibur flow cytometer with Cell Quest Pro (BD Bioscience, Franklin Lakes, NJ) and ModFit LT software (Verity, Topsham, ME). Three independent experiments with each cell line were carried out. Ploidy was estimated as the DNA content of G1 cells in the tumor cells normalized to diploid Chinese hamster ovary cells.

### Irradiation

Log phase cultures were irradiated with different doses of ^137^Cs gamma-rays using a J.L. Shepherd Model Mark I-68 nominal 6000 Ci ^137^Cs irradiator (J.L. Shepard, Carlsbad, CA), delivered at approximately 2.5 Gy/min at room temperature.

### Clonogenic Survival Assay

Radiosensitivity was measured by clonogenic assay for nonsuspension cell lines. Randomly dividing cells in T-12.5 flasks were irradiated, trypsinized and plated in triplicate onto 100 mm or 60 mm culture dishes at appropriate cell density. After incubating for 1–2 weeks to allow colony formation, dishes were rinsed with 0.9% NaCl, fixed with 100% ethanol and stained by 0.1% crystal violet. Each colony consisting of more than 50 cells was scored as a survivor. At least three independent experiments were carried out, then survival curves were drawn using linear-quadratic regression equations with Prism 5 software. The survival fraction at 2 Gy radiation (SF2) and the survival fraction at 5 Gy radiation (SF5) were obtained by interpolation of cell survival as estimates of the intrinsic radiosensitivity of each cell line.

For suspension cultures, a limiting dilution assay was used as previously utilized in human cancer cell lines [13, 14]. Cells were plated in 96-well microtiter plates at densities of 1–200 cells per well at two or three cell densities per dose point. After irradiation, the plates were incubated at 37°C for 2–3 weeks before scoring as negative or positive for growth based on microscopic examination (i.e. wells in which cell growth had occurred are positive). Based on the Poisson distribution, survival fractions were calculated as described previously [15].

### γ-H2AX Foci in G1-Irradiated Cells

Induction of, and residual DNA DSBs by IR were assessed using the γ-H2AX assay. We carried out the assay with cells synchronized and maintained in G1 during irradiation using the isoleucine deprivation method [16]. This was done because non-irradiated cells in S-phase have much higher levels of γ-H2AX foci, and also because the number of foci per cell depend on DNA content [17]. Cells were cultured for 24 hours on plastic chamber slides to approximately 50% confluence and washed with PBS once. The normal growth medium was replaced twice in 1.5 doubling times with isoleucine-deficient MEM containing 5% 3× dialyzed FBS to synchronize the cells in the G1 phase. After G1 synchronization and exposure to 0 Gy or 1 Gy of gamma-rays, cells were incubated with 5-ethynyl-2’-deoxyuridine (EdU) for 30 minutes (either immediately or after 5.5-hour incubation for repair). EdU-labeling was used to judge G1 synchronization [18]. The cells were then washed in PBS and fixed in 4% paraformaldehyde followed by permeabilization with 0.5% Triton-X 100 and 0.1% SDS. EdU was first stained as the manufacturer’s instructions. Slides were washed three times in PBS, fixed in 4% paraformaldehyde and blocked in PBS with 10% goat serum overnight at 4°C. Following overnight incubation, the cells were incubated with a phosphorylated histone H2AX antibody (Ser139) (γ-H2AX) (Millipore, Billerica, MA) and followed by Alexa 594 Fluor-conjugated goat anti-mouse antibody (Molecular Probes, Eugene, OR). The cells were mounted in a solution with DAPI containing slow fade (Invitrogen). Digital images were captured using an Axioskop motorized Z-stage Microscope (Carl Zeiss) with CoolSnapHQ2 (Photometrics, Tucson, AZ) and Metamorph software (Molecular Devices, Sunnyvale, CA). The images were used to count γ-H2AX foci per cell. Three independent experiments were carried out and numbers of the γ-H2AX were counted for a minimum of 50 EdU staining negative cells for each sample in each experiment.

### Analysis of Apoptosis

Apoptosis induction by IR was assessed using the Caspase 3/7 activation, Annexin V staining, and terminal deoxynucleotidyl transferase (TdT)-mediated deoxyuridine triphosphate nick end-labeling (TUNEL) assay. Log phase growing cells were irradiated with 0 Gy or 5 Gy gamma-rays. After 16 hours of incubation, the early apoptosis was measured with the activation of Caspase 3/7 by Caspase-Glo 3/7 kit (Promega, Madison, WI). Glow luminesce of 10,000 cells was measured by Lumat LB9507 (Berthold technologies, Oak Ridge, TN). After 24 hours of incubation, the early/late apoptosis was measured with the Annexin V staining by FITC Annexin V apoptosis detection kit with 7-AAD (Biolegend, San Diego, CA). Annnexin V positive but 7-AAD negative (early apoptotic cells) and Annexin V positive and 7-AAD positive (late stage apoptosis) was determined by Guava-PCA flow cytometer. After 48 hours of incubation, the late apoptosis was measured with TUNEL assay and fragmented nuclear morphology. The cytogentrifuged cells were fixed with 4% paraformaldehyde and ice-cold 70% ethanol. The cells were incubated in the reaction mixture containing 1.5 mM CoCl_2_, 12.5 U TdT, 1 mM 5-Bromo-2’-deoxyuridine-5’-triphosphate in TdT buffer (Br-dUTP, Sigma-Aldrich: the others, Roche, Indianapolis, IN) for 4 hours at 37°C in the dark. The slides were incubated with a mouse monoclonal BrdU antibody and Alexa 488 Fluor-conjugated goat anti-mouse antibody. Finally, the cells were counterstained with DAPI. Approximately 1,000 nuclei from each slide were counted, and TUNEL positive frequencies were calculated. Apoptosis ratios were also determined by scoring DAPI staining cells with fragmented nuclear morphology.

### Western Blotting

Cells were lysed with M-PER Mammalian Protein Extraction Reagent (Thermo Fisher Scientific) and protease inhibitors. Protein extracts (20 μg per sample) were size-fractionated on NuPage 4–12% Bis-Tris gels (Invitrogen), electro-transferred to nitrocellulose membranes (Bio-Rad) in buffer (25 mM Tris, 192 mM glycine, 20% (v/v) methanol, and 0.01% SDS) at a current density of 3.0 mA/cm^2^ for 16 hours at 4°C. The filters were blocked with Tris-buffered saline with 0.05% Tween 20 containing 2% (w/v) skim milk, and reacted with a primary antibody for 2 hours at room temperature, followed by an incubation with a secondary antibody for 1 hour at room temperature. The immunoreactive signals were detected using SuperSignal Western Blotting Detection Kit (Thermo Fisher Scientific) and a ChemiDoc XRS+System (Bio-Rad). Protein expression from band strength was analyzed by Image Lab software (Bio-Rad). The primary antibodies used in this study were the mouse anti-DNA-PKcs monoclonal antibody (Ab-4; Neomarkers, Fremont, CA), the rabbit anti-Rad51 polyclonal antibody (H-92; Santa Cruz Biotechnology Inc., Santa Cruz, CA), the rabbit anti-FANCD2 polyclonal antibody (NB100-182; Novus Biologicals, Littleton, CO) and the mouse monoclonal beta-actin antibody (Abram 8226; Abcam, Cambridge, MA). The secondary antibodies were goat anti-mouse IgG HRP conjugated antibody (1:10,000) (Santa Cruz Biotechnology, Inc., Santa Cruz, CA) and goat anti-rabbit IgG HRP conjugated antibody (1:10,000) (Cell signaling, Boston MA). Each expression band was estimated from molecular weight of each protein and the band detected in human cancer cell line A549.

### Statistical Analysis

For statistical analysis, GraphPad Prism 5 software was used. The D'Agostino-Pearson test was used to determine if the values were normally distributed. Differences with a P value of less than 0.05 were considered statistically significant. Correlations of SF2 and other parameters were determined by Pearson test. Statistical comparisons of mean values in the γ-H2AX assay (control vs 6 hours following 1 Gy) were performed using Kruskal-Wallis test followed by Dunn’s multiple comparison test. Statistical comparison of mean values in the SF2/SF5 (radioresistant group vs radiosensitive group) and in the apoptosis assay (0 Gy vs 5 Gy) was performed using unpaired two tailed t-test.

## Results

### Basic Characterization of Canine Cancer Cell Lines

Each cell line was characterized by a combination of data representing cellular doubling time, cell cycle distribution, ploidy pattern, and chromosome number, with the data summarized in Table 1. The doubling times of each cell line ranged from 15 hours (CML-6M, CTAC) to 40 hours (STSA-1), showing wide variation. We measured the cell cycle distribution of each cell line by flow cytometry. The percentage of cells in each phase of the cell cycle ranged from 39.3% (CLBL1) to 69.6% (BR) for G1 phase, from 17.3% (Parks) to 50.1% (CLBL1) for S phase, and from 1.52% (BR) to 29.9% (Parks and STSA-1) for G2/M phase. These cell lines displayed variable average numbers of chromosomes, ranging from 57 (1771) to 155 (17CM98). The canine cancer cell lines showed increased numbers of metacentric chromosomes (X-shaped chromosomes) resulting from Robertsonian translocation events [19], with the exception of two cell lines (Jones and OSW) (Table 1). Frequencies of metacentric chromosomes varied among cell lines from less than two, the normal karyotype, to 43.2 (D17) per cell. All cell lines except BR had greater than the diploid amount of DNA. We found overall agreements between abnormal ploidy and increased chromosome numbers, but not always. Nike, for example, had a smaller number of chromosome per cell with 64.4, but the ploidy pattern was between diploidy and triploidy.

### Clonogenic Survival Following Exposure to Gamma-Radiation

**Fig 1:**
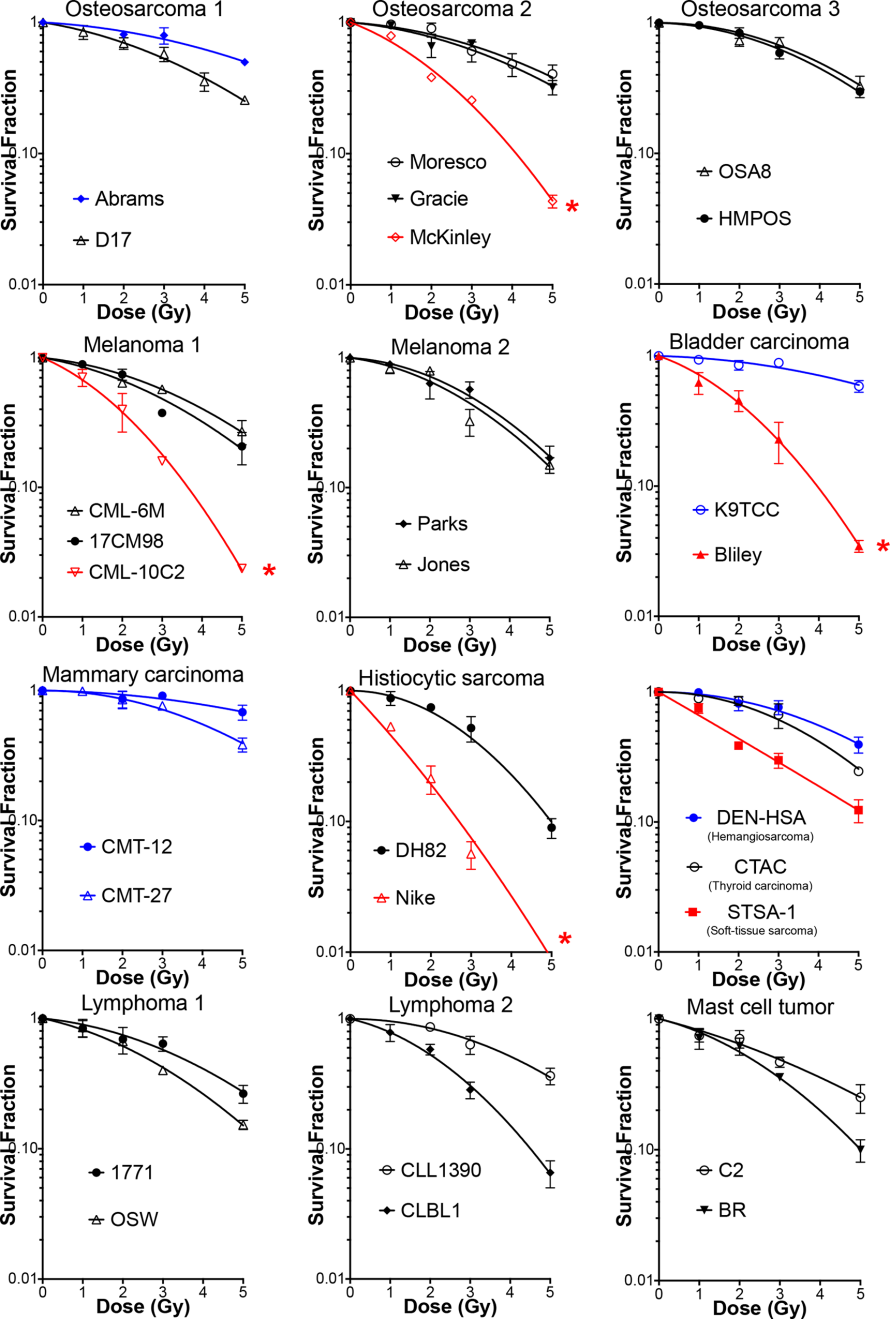
Radiation induced survival curves in 27 canine cancer cell lines. Experiments were carried out at least three times and error bars indicate the standard error of the means. We selected the most radiosensitive cell lines (red curves) and the most radioresistant cell lines (blue curves) for the following analysis.

**Fig 2:**
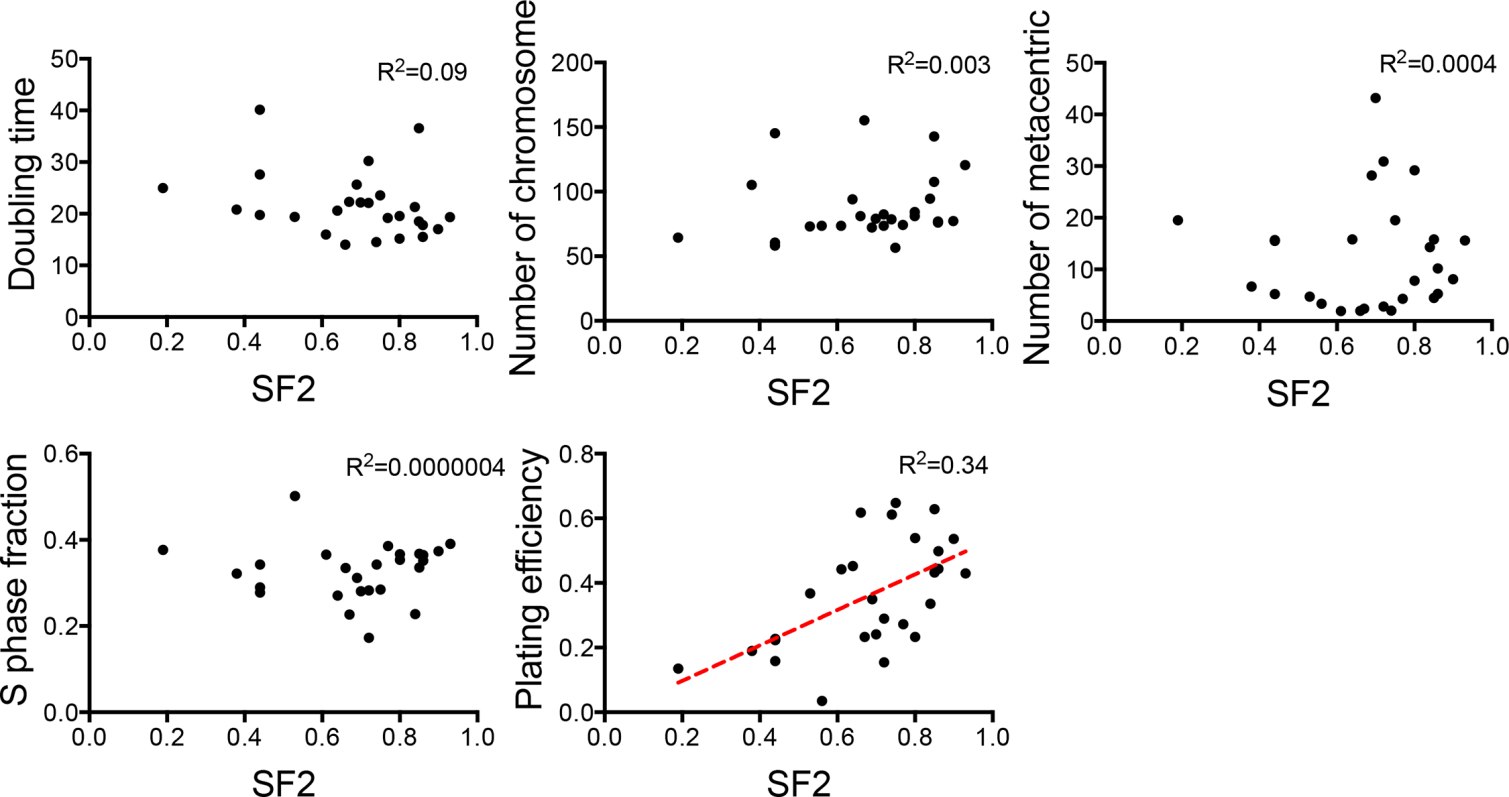
Plots of the cellular characteristics as a function of the SF2 values in each cell line. Each dot represents a cell line. The correlations were assessed using the Pearson test. The lines were fitted by a least-squares method.

We irradiated each of the 27 canine cancer cell lines with 0, 1, 2, 3 or 5 Gy of gamma-rays and measured colony formation. Their survival curves are shown in Fig 1. SF2 and SF5 values that were calculated from the linear quadratic regression survival curve, as well as plating efficiency, are reported in Table 1. These data represent the range of canine tumor cell radiosensitivities across different tumor types. The SF2 ranged from 0.19 to 0.93, and the SF5 ranged from 0.01 to 0.60. The plating efficiency of these cell lines also demonstrated a wide variation and ranged from 4% to 65%. The plating efficiency of BR (4%) was too low to obtain reliable survival fractions; therefore, its radiosensitivity was not used for further analyses. The radiosensitivity of the four canine OSA cell lines (D17, Moresco, Gracie and MacKinley) measured by clonogenic assay was consistent with those that have previously been published [20]. Therefore, the data of the basic cellular characteristics (e.g. doubling time, chromosome analysis) shown in the report were also used for the analysis in this study. We evaluated correlations between the variable cellular characteristics and the radiosensitivity. In these cell lines, there was no significant correlation of SF2 with S-phase fraction, doubling time, chromosome number, or number of metacentric chromosomes, while there was a modest but statistically significant correlation between SF2 and plating efficiency (R^2^ = 0.34, p = 0.002, Pearson test) (Fig 2). Evaluation against SF5 showed the similar results to these obtained from SF2 (R^2^ = 0.24, p = 0.01, Pearson test).

### Selection of Radiosensitive and Radioresistant Groups

**Fig 3:**
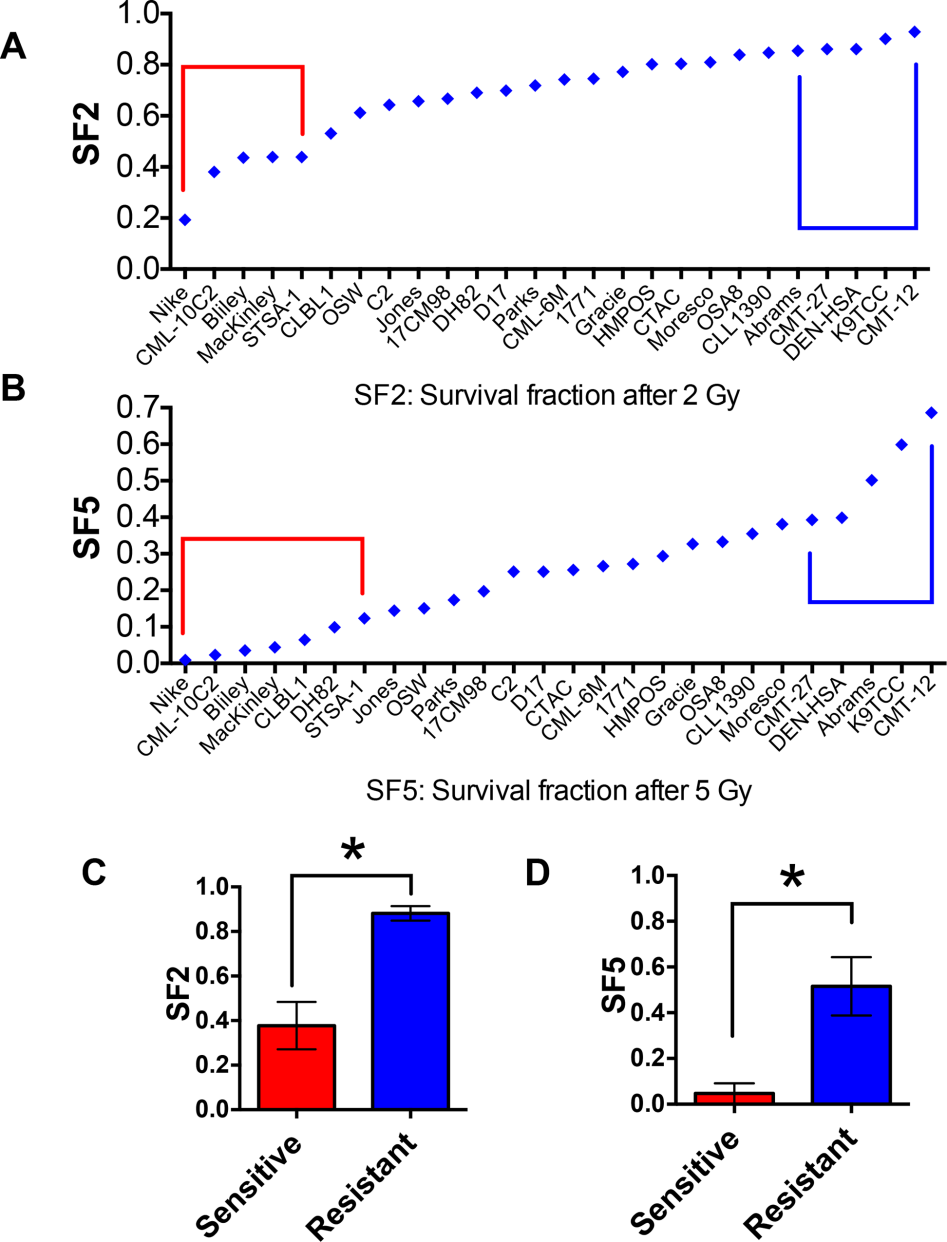
The measured SF2 and SF5 of 27 canine tumor cell lines. 27 cell lines are ranked based on SF2 (A) and SF5 (B). Red circle: radiosensitive group, blue circle: radioresistant group. (C, D) Comparison between the mean values of two groups based on the SF2 (C) or SF5 (D). Error bars indicate standard deviation. * p<0.001 versus sensitive and resistant group (unpaired t-test).

The 27 canine cancer cell lines were ranked by the radiosensitivity parameters, SF2 and SF5, and the five most sensitive or resistant lines for each parameter were defined as the sensitive and resistant groups (Fig 3A and 3B). The selected radioresistant group displayed SF2 values from 0.19 to 0.44 (Nike, CML-C2, Bliley, MacKinley, STSA-1). The radiosensitive group exhibited SF2 values above 0.86 (CMT-12, K9TCC, DEN-HSA, CMT-27, Abrams). The differences in tumor cell radiosensitivity were better discriminated when the radiosensitivity of tumor cells was expressed as survival fractions at the higher dose (SF5). The cell lines in the two groups based on the SF5 comparison were slightly different from those based on the SF2 comparison. The survival fractions between two groups of cell lines based on either the SF2 or SF5 comparison were statistically different (p<0.001) (Fig 3C and 3D).

### Relationship between Intrinsic Radiosensitivity and DNA DSBs in G1-Irradiatied Cells

**Fig 4:**
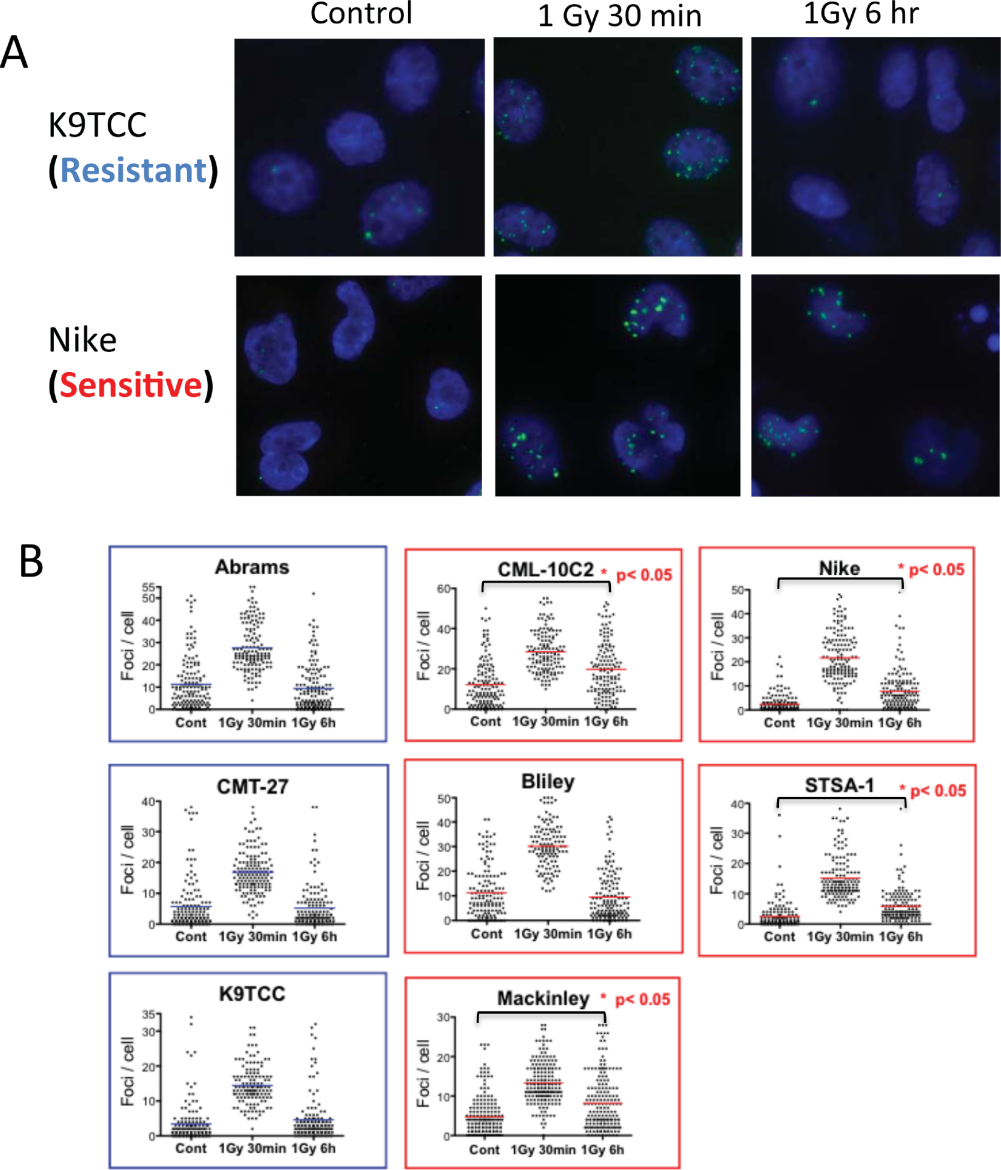
Phosphorylated-H2AX foci in G1 irradiated cells of radioresistant and radiosensitive groups of canine tumor cell lines. Cells were synchronized in G1 using isoleucine deficient media and then irradiated with 1 Gy of gamma-rays. Following 30 min or 6 hr incubation time, cells were stained with γ-H2AX. (A) Examples of γ-H2AX foci (green) in nuclear DAPI (blue) staining in control, 1Gy followed by 30 min incubation and 1 Gy followed by 6 hr incubation in EdU negative cells. (B) Quantitative analysis of gamma-H2AX foci per cell. The pooled data from three independent experiments scoring 50 cells in each experiment are shown. The bar indicates mean. Statistical significances are shown for only control versus 6 h after 1 Gy (nonparametric Kruskal-Wallis test).

To examine whether the response of cells to DNA DSBs correlates with radiosensitivity in the canine cancer cell lines, we used γ-H2AX assay in the five most radioresistant and five most radiosensitive cell lines selected from SF2 ranking. Nuclear foci of phosphorylated histone H2AX (γ-H2AX) resulting from DNA damage are sensitive markers for DNA DSBs [21]. We measured the number of γ-H2AX foci after 1 Gy gamma irradiation in G1-phase synchronized cells following 30-min or 6-hour repair time (Fig 4). In the isoleucine deficient media to synchronize cells in G1, most DEN-HSA cells died after a 24-hour incubation, and the CMT-12 cell line was not synchronized in the media. Therefore, these two cell lines were not used for this analysis. The other cell lines showed G1 synchronization with less than 16% in the S-phase in the isoleucine deficient media for 1.5 doubling times. In some cells not in S phase with 0 Gy treatment, large numbers of endogenous γ-H2AX foci were observed in all of the canine cancer cells. We excluded cells with high levels of foci outside of IR-induced distribution from the analysis to detect the levels of foci induced by IR. Based on the analysis without cells with high levels of endogenous γ-H2AX foci numbers, 1 Gy of gamma-rays induced significantly higher levels of γ-H2AX foci after 30 min irradiation in all cell lines utilized in this analysis (p<0.05 vs 0 Gy, not shown in the figure) (Fig 4B). At 30 minutes after irradiation, the median number of γ-H2AX foci was dependent on the DNA content of each cell line. Furthermore, the radiosensitive CML-10C2, Nike, STSA-1 and MacKinley had higher numbers of γ-H2AX foci after 6 hour following 1 Gy irradiation compared to their control levels (p<0.05 vs 0 Gy). On the other hand, all three of the radioresistant cell lines and one radiosensitive cell line, Bliley, didn’t show significant differences between control cells and cells with 6 hours following irradiation.

### Relationship Between Intrinsic Radiosensitivity and Apoptosis Frequency in Irradiated Cells

**Fig 5:**
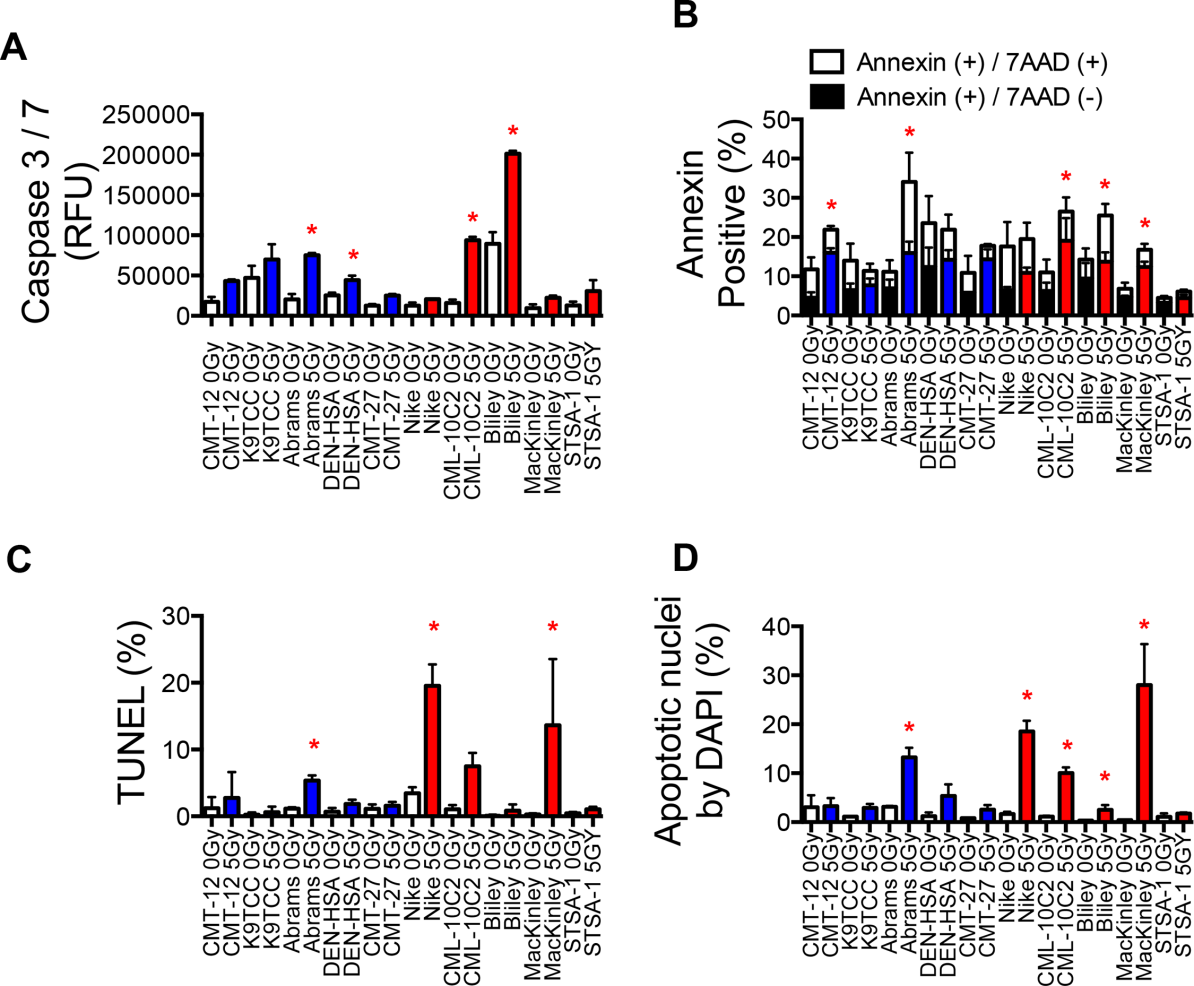
Radiation-induced apoptosis in the radioresistant and radiosensitive canine cancer cell lines. (A) Caspase 3/7 activity measured by luminomator at 16 hours after irradiation. (B) Annexin V and 7AAD staining measured by flow cytometer at 24 hours after irradiation. (C) TUNEL staining and (D) DAPI staining measured by fluorescent microscope. Cells irradiated with 0 Gy and 5 Gy of gamma-rays. *, p<0.05 versus 0 Gy and 5 Gy for each cell line (unpaired t-test).

The five radioresistant cell lines and the five radiosensitive cell lines were also assessed for apoptosis at 18, 24, and 48 hours post-irradiation (0 Gy and 5 Gy) (Fig 5). Caspase 3/7 activation was analyzed 18 hours post-irradiation (Fig 5A). Annexin V expression was analyzed at 24 hours post-irradiation (Fig 5B). The activity of Caspase 3/7 increased in more than two times were observed in CMT-12, Abrams, CMT-27, CML-10C2, Bliley, MacKinly, and STSA-1. The percentage of apoptotic cells increased significantly with 5 Gy irradiation in CMT-12, Abrams, CMT-27, CML-10C2, Bliley, and MacKinley by Annexin V analysis. Apoptotic cells were counted by TUNEL staining (Fig 5C) and DAPI stating (Fig 5D) and reported separately. Although apoptosis was seen in all control cultures ranging 0.1–3.4% by TUNEL staining and 0.37–3.3% by DAPI staining of the cells, depending on the cell line, there was no significant difference between the cell lines. The percentage of apoptotic cells increased significantly with 5 Gy irradiation in Abrams, Nike, and CML-10C2 by DAPI staining relative to 0 Gy samples (p<0.05, unpaired t-tests) (Fig 5C). Using the measurement of apoptotic cells by TUNEL assay, similar results to these by DAPI staining were observed. A significant increase in apoptosis frequency by IR were observed in one (Abrams) out of the five radioresistant cell lines and in three (Nike, CML-10C2 and MacKinley) out of the five radiosensitive cell lines (Fig 5D).

### Protein Expression of DNA Repair pathway in Radiosensitive and Radioresistant Groups

**Fig 6:**
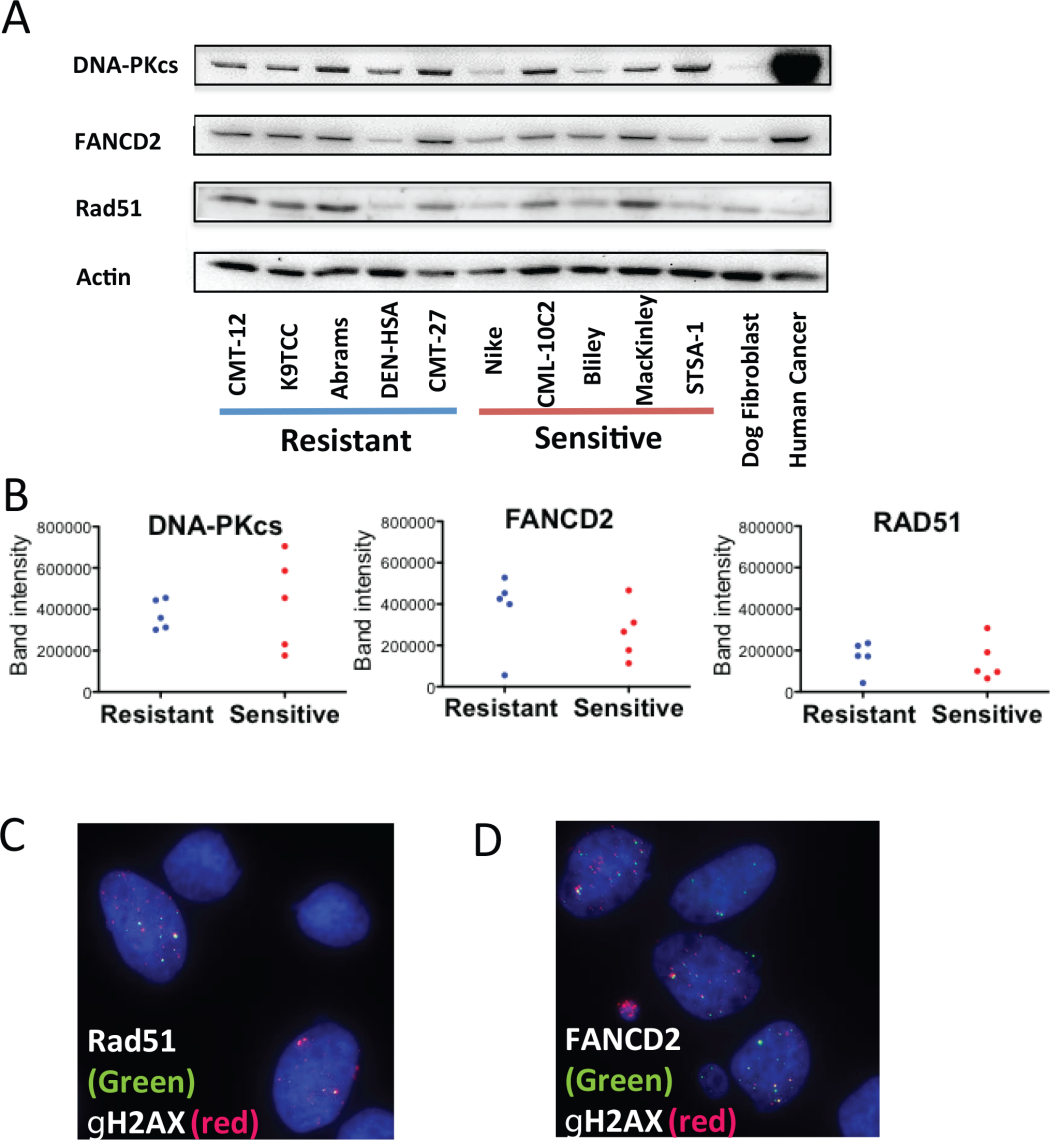
Basal expressions of DNA repair proteins in the radioresistant and radiosensitive canine cancer cell lines. (A) Western blot analysis of DNA-PKcs (460 kDa), FANCD2 (165 kDa) and RAD51 (37 kDa). β-actin (42 kDa) expression was used as a control. Each expression band was estimated from molecular weight. (B) Band intensity of western blot. (C, D) Representative images for RAD51 foci (C) and FANCD2 foci (D) co-localized with gamma-H2AX in Moresco cells.

Since DNA DSB repair pathways are closely related to cell killing by IR, we studied the protein status of the major pathways in the canine cancer cells. We focused on the two major non homologous end joining (NHEJ) and homologous recombination (HR) in the DSB repair and Fanconi Anemia (FA) pathway, which possibly contributes to DNA damage repair for irradiation. Protein expression of major players in each pathway, DNA-PKcs in NHEJ, RAD51 in HR and FANCD2 in FA pathway were detected by western blotting in the radioresistant and radiosensitive groups (Fig 6). The expression of DNA-PKcs, FANCD2 and RAD51 were not uniform among the cell lines, and there was no clear trend between the expression levels in the two groups as shown in Fig 6B. The expression of the three proteins in the canine cancer cells were overall higher than those in normal dog fibroblasts. We observed less expression of DNA-PKcs proteins in Nike (1.4 fold of normal) and the highest expression in STSA-1 (5.4 fold of normal). For the FANCD2 protein, the lowest expression was in DEN-HSA (0.8 fold of normal) and the highest expression was in CMT-27 (7.2 fold of normal). For the RAD51 protein, the lowest expression in DEN-HSA (0.4 fold of normal) and the highest expression was in MacKinley (3.0 fold of normal). When compared between the canine and the human cancer cell line (A549), the expression levels of DNA-PKcs in STSA-1, which showed the highest expression of the canine cell lines, were 10 times less than that of A549.

We also observed the foci of RAD51 and FANCD2 as functionally co-localized with γ-H2AX on the replication stress without irradiation in all 27 cell lines (Fig 6C and 6D). Another advantage for testing RAD51 and FANCD2 was that these protein foci formations require other upstream proteins, such as five RAD51 paralog proteins and eight Fanconi anemia proteins [22–24]. Therefore, detecting foci formation enabled us to screen for the presence of these upstream proteins in all the 27 canine cancer cell lines. We observed functional foci of RAD51 and FANCD2 in all of the cell lines.

## Discussion

The 27 canine cancer cell lines derived from ten different tumor types utilized in the present study had varying radiosensitivities regardless tumor type with the SF2 values of 0.17 to 0.94 (Fig 1 and Table 1). From previous radiosensitivity data using a wide variety of human tumors, SF2 ranged from 0.038–0.95 [13]. The lower range of SF2 reported in the human study was mostly due to lymphoid tumors, which were not highly radiosensitive in our canine result where SF2 was measured using the same assay. Lymphoid tumors are known to be sensitive to radiation therapy in both human and veterinary oncology [2]. This discrepancy might be caused by the variation between lymphoid tumor cell lines as reported previously [25] since only four cell lines were investigated in our study.

In order to understand parameters that might contribute to intrinsic radiosensitivity, we evaluated the relationships of cellular radiosensitivity with basic cellular characteristics in the 27 canine cancer cell lines. A wide variation was also observed in cellular characteristics in terms of chromosome number, S-phase fraction, doubling time and plating efficiency in these cell lines (Table 1). In our study, we didn’t find a correlation between the radiosensitivity and the cellular characteristics including chromosome number, S-phase fraction, and doubling time (Fig 2). Previously, the largest number of chromosomes has been found in some of radioresistant human cancer cells [9]. It is possible that such lines can better tolerate chromosome loss because of a greater degree of genomic redundancy. However, this is not a consistent observation in human cancer cell lines [26]. Since S-phase cells are known to the most radioresistant in the cell cycle, fraction of S-phase in cell population is possible a parameter of radiosensitivity in cancer. However, some human studies have not shown correlations [27] and some have shown [10]. In human studies, cell proliferation rates are known as one of the important parameters mainly in tissues *in vivo* [5, 28]. Therefore, based on the uncertainty of these parameters, our results in canine cancer cell lines agreed with the human studies. In contrast, plating efficiency of the cells in the 27 cell lines showed modest correlation with radiosensitivity in our study (Fig 2). This parameter has not been discussed in depth in previous papers, except where one report noted that resistant cells had better plating efficiencies [27] and another study reported no correlation [28]. However, some canine cell lines with low plating efficiency (Moresco, Gracie and Parks) showed radioresistance; therefore, plating efficiency was not perfectly related to radioresistance (Table 1 and Fig 2). Plating efficiency may be potentially associated with cancer stem cell population in each cell line. We would need to evaluate the plating efficiency in an additional panel of canine cancer cell lines to confirm whether this is a more prominent feature in dogs.

In the present study, the known parameter DNA DSB repair efficiency following exposure was evaluated between radiosensitive and radioresistant cell lines. The cell lines from the two groups based on the SF5 comparison were only slightly different from those based on the SF2 comparison (Fig 3A and 3B), therefore, we used the cell lines selected by the SF2 comparison. DSB repair is an obvious candidate marker for radiation response because DSBs are considered the most critical type of DNA damage caused by IR. In normal cells, both slower overall rates of DSB rejoining and higher levels of residual breaks are associated with more radiosensitive phenotypes [29]. In this study, we used the γ-H2AX assay, which is known to be a sensitive method to measure DSBs [21]. In G1-synchronized cells, the high levels of residual γ-H2AX foci were observed in the four out of the five radiosensitive canine cancer cell lines (Fig 4B). In human cancer cell lines, the relationship of residual levels of γ-H2AX or rates of its disappearance to clonogenic cell survival in cancer cells has been studied by several investigators, and correlations were found in some studies [30, 31], but not all [32]. This discrepancy might be due to the high endogenous γ-H2AX foci in cancer cells relative to normal cells as previously observed [33], which was also observed in canine cancer cells. These high endogenous γ-H2AX foci in non-irradiated cells were not in the S-phase cells where γ-H2AX foci occur in cell nuclei during replication stress [34]. We decided to exclude these cells with foci numbers outside of the distribution induced by IR because the high background level possibly obscures the γ-H2AX foci change by repair. The development of protocols to better cut out high levels of endogenous γ-H2AX foci may help the analysis to explore DNA DSB repair as a robust parameter to determine responses of tumor cells to radiation. On the other hand, isoleucine depletion induced G1 phase synchronization could not hold cells in G1 phase for 24 hours. Our analysis was limited to 6 hours repair time. Furthermore, as reported in previous studies using human tumor cell lines [30, 32], analysis of γ-H2AX using flow cytometry might be a better method to evaluate radiosensitivity of canine cancer cells.

Apoptosis has been studied as an important element that determines radioresponsiveness of cancer cells, especially lymphoid tumor cells [11]. Its correlations with radiosensitivity have been variable in previous human studies using solid tumors [4]. In our study, the percentage of cells that underwent apoptosis at 16 to 48 hours following 5 Gy differed considerably between the canine cancer cell lines (Fig 5). However, the apoptosis frequency didn’t clearly distinguish two groups (radiosensitive and radioresistant), suggesting incomplete parameters like in human cancer. We used ten different tumor types of 27 canine cell lines in our study. It is possible radiation induced apoptosis and intrinsic radiosensitivity may be clear when same tumor type is compared [35]. Apoptosis is just one of modes of radiation induced cell death [36]. We did not evaluate G1-block [37], senescence [36], and autophagy [38]. Clonogenic cell survival evaluates sum of any types of cell death. Further research will be needed to characterize the cell death modes fully.

The expression and function of radiation damage associated proteins were also analyzed to investigate intrinsic radiosensitivity. DNA-PKcs expression, which highly associates with cellular radiosensitivity [39], varied in each cell line but not clearly distinguished radiosensitive group and radioresistant group. Loss of HR repair or FA pathway is associated with some tumors and leads radiosensitivity but not severe as loss of NHEJ does [40]. Although we tested two DNA DSB repair pathways and FA pathway, expression or functions of these pathways didn’t clearly distinguish two groups. Background level of caspase 3/7 activity was also analyzed to evaluate potential activity for apoptosis. It didn't distinguish radiosensitive and radioresistant groups.

In summary, our results indicated that these canine cancer cell lines have a wide variation regarding radiosensitivity and basic cellular characteristics. IR-induced DNA DSB repair was related to radiosensitivity, which is consistent with human study. These data may assist further investigations using the canine cancer cell lines to explore the underlying mechanisms of intrinsic radiosensitivity of cancer. Especially, further assessment focusing on the detection of DNA DSB should involve predicting individual response to RT, regardless of tumor type.
